# Continuity of health service delivery during the COVID-19 pandemic: the role of digital health technologies in Uganda

**DOI:** 10.11604/pamj.supp.2020.35.2.23115

**Published:** 2020-05-20

**Authors:** Louis Henry Kamulegeya, John Mark Bwanika, Davis Musinguzi, Pauline Bakibinga

**Affiliations:** 1 The Medical Concierge Group, Kampala, Uganda; 2 African Population and Health Research center, Nairobi, Kenya

**Keywords:** COVID-19, telehealth, telemedicine, Uganda, digital health

## Abstract

In response to coronavirus disease-2019 pandemic (COVID-19), the government of Uganda instituted movement restrictions to curb disease spread. However, this affected accessibility to medical services in a setting where the healthcare system is not equipped to handle most healthcare needs of the populace outside hospital premises. This gap led to the prominence and unprecedented rise in the use of digital health technologies to deliver health information and services at a distance (telehealth) during the COVID-19 outbreak. The use of telehealth modalities including tele-consultation, tele-psychiatry, call centers and mobile phone health information dissemination increased. The COVID-19 pandemic augmented the rising role of digital health technologies as a much needed aspect of medical service delivery in our times. However, the efficacy and impact on clinical outcomes across various healthcare thematic areas need to be explored further and more evidence generated.

## Commentary

Following the declaration of COVID-19 as a global pandemic, countries globally and in sub-Saharan Africa, including Uganda instituted lockdown measures in various degrees. Such measures included movements restrictions and curfews unless following a protracted approval process from political leaders in one’s locality. These measures resulted in a disruption of healthcare service access for both acute and pre-existing medical conditions [1]. The lockdown measures have had a negative impact at population level, in terms of continued access to basic human needs like food and health care services. Reports of patients with chronic illnesses missing hospital appointments in fear of contracting the disease from contact spread have been reported in different parts of Uganda [2]. Hospitals are perceived by many members of the public as hotspots for COVID-19 spread and many individuals struggle to find health care plans due to the limitations of cost and availability. As a result, there are reports of limited health access, especially among vulnerable groups like people living with HIV/AIDS (PLHIV), pregnant women and children among others [2]. In high income settings, digital health technologies have been harnessed effectively during this period with examples including tele-consultations, artificial intelligence supported symptom checker applications among others [3,4]. People were encouraged to call or use mobile applications with their primary health caregivers first. Such actions build on the growing evidence for the value of digital health enabled medical consulting in reducing physical and financial access barriers as well as protecting health workers and patients from infections. Below, we summarize the range of telehealth centered interventions that were deployed to support the continuity of routine health care services during the COVID-19 outbreak in Uganda as of end of April 2020.

Telehealth in Uganda before COVID-19: telehealth is a broad term referring to the use of digital health technologies to remotely deliver clinical and non-clinical services including provider training, administrative meetings and continuing medical education. While telemedicine is specific to the delivery of health care services at a distance using information and communication technologies to support and enable long-distance patient care, maintenance of patient health record and provision of patients and professional health services [5]. Before the COVID-19 pandemic, access to healthcare services was mainly through the traditional modality with patients queuing up at physical health facilities for medical consultations, access to laboratory or medicines. However, early adopters of telehealth services in Uganda like the medical concierge group (TMCG), infectious diseases institute (IDI) and Baylor Uganda supported a number of cross cutting areas including HIV/AIDS, tuberculosis, maternal newborn and child health, sexual and reproductive health services among others. Majority of these telehealth interventions have been part of a wider donor funded public health project for example; the USAID-HIV/AIDS initiatives at workplaces activity, USAID regional health integration to enhance services (RHITES), North Lango among other health system strengthening projects. The telehealth services availed included; remote tele-consultations via voice, chat and video platforms, SMS reminders on facility appointments and mobile SMS health information dissemination and awareness for behavioral change [6]. Other telehealth modalities like interactive voice recordings and artificial intelligence powered self-triage applications have also been implemented in the pre-COVID-19 era.

### Application of telehealth during COVID-19 in Uganda

#### Tele-consultations

Using mobile phone applications like short messaging service (SMS) and voice calling among other applications, beneficiaries have been able to remotely consult with healthcare providers. This has helped patient triage and reduced chances of health facility congestion in the face of easy spread of COVID-19. Teleconsultation providers during the COVID-19 pandemic included private entities like Rocket Health, Twogere Health, Seven Doctors, GoGP+ and academic research institutions like infectious diseases institute and Baylor Uganda among others. Health facilities that previously leveraged traditional physical consultations have adopted the teleconsultation model during the pandemic. Examples of such facilities included Nakasero hospital, Case hospital and UMC Victoria hospital all availed different platforms for their beneficiaries to tele consult with healthcare providers (Table 1).

**Table 1:** teleconsultation providers during the COVID-19 pandemic in Uganda

Early Adopters	
**Name of Organization**	**Teleconsultation channels used**
**Early Adopters**	
*Rocket Health, The Medical Concierge Group	Chat, voice calls, video, WhatsApp
***Ministry of Health, Uganda	Voice call
~Infectious Diseases Institute	Voice call
~Baylor Uganda	Voice call
**Late Adopters**	
*Twogere Health	Smart-phone Application
*Seven Doctors	Smart-phone Application
*GoGP+	Smart-phone Application
**Nakasero Hospital	Voice call and Video
**Case Hospital	Voice call and WhatsApp
**UMC Victoria	Voice call
***Kampala Capital City Authority	Voice call

* Private company ** Physical health facility *** Public services ~Academic Research Institutions

**Figure 1:**
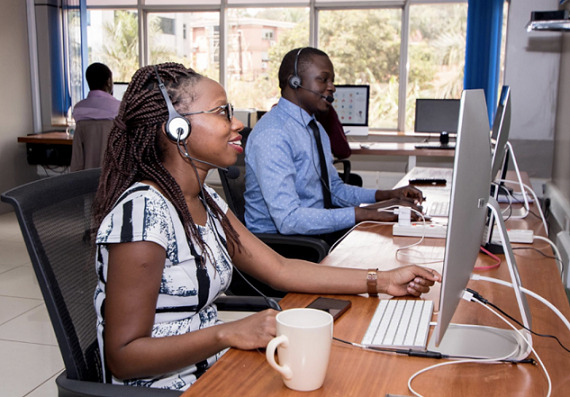
Rocket health call center in use during COVID-19 outbreak

**Call centers and online health:** Figure 1 shows the Rocket Health call center in use during COVID-19 outbreak. Other call-center service providers that have been made available during the COVID-19 pandemic included ministry of health, infectious diseases institute, Baylor Uganda and the Kampala Capital City Authority call centers. The call centers are currently used as a source of health information to the general public, in addition to providing triage and referral services to callers. Online search platforms that enable easy access to health service points are also available to the general population.

**Mobile phone health information dissemination:** health content on COVID-19 has been disseminated via mobile SMS by different telecommunication companies and partners in support of the ministry of health. This health content covered different thematic areas including disease awareness creation and prevention measures. The content was made available only in english language. Below is an example of such a mobile message: *“Do you know that Coronavirus can spread from person to person? Avoid crowded places and direct contact with people through handshakes”.*

**Telepsychiatry:** mental health support services including tele counselling and telepsychiatry were notably on demand during the pandemic owing to the rise in vices like gender-based violence. Non-government organizations like Reach A Hand availed toll-free hotline for the population. Resilience Africa network (RAN) a research institution based out of Makerere University supported a smart phone-based application called Centers4Her that availed sexual and reproductive health services like Tele counselling, among others, during the COVID-19 outbreak for the Ugandan population.

**Figure 2:**
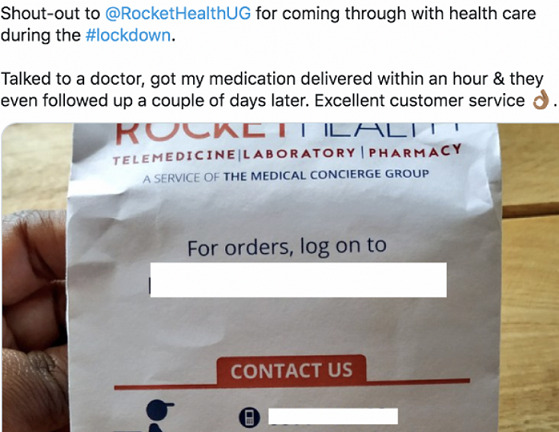
Client feedback experience from utilizing the mobile medical services during COVID-19 lockdown

Mobile medical services (tele-laboratory & tele-pharmacy): health service providers innovated around the lockdown to ensure continuity to accessing the much-needed medical services like the laboratory and medicine refills. Medicine delivery services that involved providers taking the prescription refills of clients to their preferred locations (home) were on a rise. In addition, mobile laboratory services involving providers picking specimen/samples from client’s location (home) were noted. These services came off as very convenient and helped address the limitations created by movement restrictions during the COVID-19 pandemic to ensure continuity in health service delivery. Additional medical services that were delivered at client’s homes included; infant immunization. Figure 2 shows one of the client feedbacks from using the mobile medical services during the COVID-19 lockdown.

### Benefits of telehealth

The COVID-19 pandemic has expanded the use of telemedicine and other technologies in delivery of healthcare services in Uganda. Such trends augment the growing evidence of the effectiveness of telemedicine approaches especially in the areas of teleconsultations, health information dissemination and telepsychiatry [7]. These platforms overcome the challenges posed by COVID-19 pandemic in accessing medical services without increasing risk of infection. The use of online platforms like social media has grown exponentially in Uganda, with the general public relying on platforms like Facebook, Twitter among others to access information from government authorities. The ministry of health capitalized on this opportunity by leveraging online platforms to share information regarding COVID-19 including routine updates. This has helped to reach a large critical mass while maintaining the social distancing. The adoption of teleconsultation by private traditional healthcare providers is evidence of the effectiveness of teleconsultation in managing outpatient medical complaints if diagnostic and therapeutic protocols are followed. Therefore, improving this modality of provider-patient interaction, telemedicine can help improve the appropriateness of hospital admissions and referrals to the specialist’s services, of the request of diagnostic tests and of chronic disease home management [8].

#### Challenges encountered in delivering telehealth services

Despite the fact that telehealth has enabled bridging the gap of continued access to healthcare services during the COVID-19 pandemic in Uganda, there were some noted challenges including: radius of service coverage: there were geographical limitations in accessing the on-demand medicine deliveries and mobile laboratory sample pick-up for non-COVID-19 related medical services as major providers were limited to Kampala. This greatly affected the completion of referrals and linkages to services uptake after the tele-consultation; documentation: especially for the late adopters that lacked proper electronic medical records systems to support tele-consultations; local language support: the outbreak has found an unprepared social behavioral change eco-system in regards to availability of health information for public dissemination. As a result, the majority of the rapidly developed content is only availed in English including the mobile SMS awareness messages. This ultimately must have an impact on the effectiveness of these messages within the less educated demographics. However, there is no evidence on this impact; costs of internet subscriptions: as some of the tele-health channels and health information platforms leveraged on third party operators like WhatsApp, Facebook etc. the extra charges incurred on over the top (OTT) tax [9] affected the ease of tele-consultation and health information access for the population; health service providers’ skills: without locally defined training programs for health service providers, it is still unknown what training arrangements those delivering telehealth services are using. More national guidelines grounded in local context are needed on this.

**Recommendations:** there is growing development of new innovations in the space of digital health, however the need to harmonize these interventions so as to avoid duplication and increase impact in a setting where resources are limited is needed. Uganda has not yet developed laws and regulations on telemedicine use yet the technology and its adoption seem to be growing at a very fast pace; there is need for the different health sector stakeholders including; Ministry of Health, Ministry of Education, health practitioners’ bodies like the Uganda Medical and Dental Practitioner’s Council, Uganda Allied Health Professional’s Council, Pharmaceutical Society of Uganda and others to recognize telemedicine and establish operation models. This will help streamline telemedicine as a medical service delivery model, prepare current and future health providers to offer telehealth services and also protect the rights and safety of the patients; as the country works towards achieving the sustainable development goal health for all strategy, affordable and easy to scale modalities of health services delivery more so those that support primary healthcare like telemedicine approaches should be implemented.

## Conclusion

It is now clear that there is a growing appreciation and rise in utilization of digital health services in Uganda. However, efficacy and impact on clinical outcomes across various healthcare thematic areas need to be explored further and more evidence generated. In addition, there is need for more evidence as to whether uptake of these services reduces demand on public health care systems.

## Competing interests

The author declares no competing interests.
